# Optimizing Cardiovascular Risk Management in Primary Care Using a Personalized eCoach Solution Enhanced by an Artificial Intelligence–Driven Clinical Prediction Model: Protocol from the Coronary Artery Disease Risk Estimation and Early Detection Consortium

**DOI:** 10.2196/66068

**Published:** 2025-08-08

**Authors:** Rutger van Mierlo, Bart Scheenstra, Joost Verbeek, Anke Bruninx, Petros Kalendralis, Inigo Bermejo, Andre Dekker, Arnoud van 't Hof, Marieke Spreeuwenberg, Laura Hochstenbach

**Affiliations:** 1 Department of Cardiology Cardiovascular Research Institute Maastricht (CARIM) Maastricht University Maastricht The Netherlands; 2 Department of Radiation Oncology (Maastro) Research Institute for Oncology and Reproduction (GROW) Maastricht University Maastricht The Netherlands; 3 Department of Cardiology Zuyderland Medisch Centrum Heerlen The Netherlands; 4 Department of Cardiothoracic Surgery Heart and Vascular Center Maastricht University Medical Center (MUMC+) Maastricht The Netherlands; 5 Department of Health Services Research Care and Public Health Research Institute (CAPHRI) Maastricht University Maastricht The Netherlands; 6 Department of Cardiology Maastricht University Medical Centre Maastricht The Netherlands

**Keywords:** atherosclerotic cardiovascular disease, disease management, cardiovascular risk management, digital health, clinical prediction model, artificial intelligence, medical informatics, cardiovascular, primary care, risk management, AI-driven, prediction model, atherosclerosis, costs, cardiovascular disease, effectiveness, eCoach

## Abstract

**Background:**

Atherosclerotic cardiovascular disease poses a heavy burden on the population’s health and health care costs. Identifying apparently healthy individuals at risk of developing cardiovascular diseases using clinical prediction models raises awareness, facilitates shared decision-making, and supports tailored management of disease prevention. In the CARRIER project, a personalized cardiovascular risk management (CVRM) eCoach approach is cocreated, in which identified individuals receive education, guidance, and monitoring to prevent atherosclerotic cardiovascular disease through existing interventions. In this approach, an artificial intelligence–driven clinical prediction model calculates the 10-year risk for atherosclerotic cardiovascular disease, which supports informed decision-making.

**Objective:**

This study aims to assess the effectiveness of our CVRM eCoach approach through a 10-year risk calculation of atherosclerotic cardiovascular disease, including risk factors contributing to this risk.

**Methods:**

This pretest-posttest interventional study provides the CVRM eCoach approach for 6 months to 100 apparently healthy individuals eligible for CVRM. The CVRM eCoach approach is a multicomponent eHealth solution, including a clinical prediction under intervention model that not only calculates the 10-year risk of cardiovascular disease through conventional risk factors (smoking, blood pressure, and lipid profile) and individual characteristics (age, gender, socioeconomic status, physical activity, and diet) but also calculates how the risk changes after hypothetical lifestyle or medical interventions. The CVRM eCoach approach includes features that encourage behavior change. Most of these features include goal setting, decision cards to help decide on an intervention, intervention monitoring, remote communication, and education, all accessible from one dashboard. A practice nurse or physician consults the individuals after risk calculation with the clinical prediction model and uses behavior change features, such as the decision cards, to support shared decision-making. Data are primarily collected via the eCoach, after which the 10-year risk for atherosclerotic cardiovascular disease and its components are analyzed using paired-sample analyses.

**Results:**

Recruitment began in March 2024 and will continue until 100 participants have been recruited, which is expected in 2025.

**Conclusions:**

We anticipate that our CVRM eCoach approach will be valuable in the primary prevention setting. During the crucial initial first months of habit formation, factors such as education, regular check-ups via the eCoach, and clear risk communication could support individuals in sustaining their medical or lifestyle interventions. We hypothesize that there will be a slight to moderate reduction in the 10-year risk of atherosclerotic cardiovascular disease, which over time will lead to significant health improvements on a larger scale.

**Trial Registration:**

CCMO NL84584.096.23; https://onderzoekmetmensen.nl/nl/trial/56578

## Introduction

Atherosclerotic cardiovascular disease (ASCVD) imposes a significant burden on both the general population and health care expenditure [1]. The risk of ASCVD is strongly associated with demographic factors such as age and sex, in conjunction with conventional risk factors such as hypertension [2], hypercholesterolemia [3], and smoking status [4]. Furthermore, nonconventional risk factors, including socioeconomic status [5], as well as lifestyle factors such as diet [6] and physical activity [7], are pivotal in the progression of ASCVD. Adhering to lifestyle recommendations substantially reduces the risk of ASCVD [8]. Nonetheless, the prevalence of modifiable risk factors remains alarmingly high [1].

Cardiovascular risk management (CVRM) aims to mitigate the impact of ASCVD at various levels, namely in individuals, specific groups with elevated risk, or at a population level. In the Netherlands, CVRM is commonly conducted in a general practitioner’s (GP’s) office and largely managed by practice nurses. The target population is apparently healthy individuals who are at heightened risk for ASCVD due to the presence of risk factors but without established ASCVD or diabetes mellitus. According to international guidelines, CVRM initiates with a personalized risk assessment to estimate the likelihood of future cardiovascular events [9,10]. Clinical prediction models (CPMs), such as the Framingham model [11] and the SCORE2 (Systematic Coronary Risk Evaluation 2) Working Group and European Society of Cardiology Cardiovascular Risk Collaboration algorithms [12,13], assist in risk assessment by estimating individualized risks for future cardiovascular events as well as the impact of lowering these risk factors. However, lifestyle factors, such as physical activity and dietary habits, are not yet included.

The Dutch CARRIER (Coronary Artery Disease: Risk Estimations and Interventions for Prevention and Early Detection) consortium is dedicated to reducing the burden of ASCVD by empowering apparently healthy individuals to adopt healthier lifestyles and mitigate their cardiovascular risk [14]. The consortium’s objectives include (1) addressing ethical and legal requirements for the use of data from different sources for the CPM; (2) creating a CPM that incorporates both conventional and specific, attainable nonconventional risk factors; (3) cocreating with health care professionals and patients to realize a personalized artificial intelligence–driven eHealth solution (eCoach) in the CVRM health care plan; and (4) evaluating the effectiveness of this eHealth solution in primary care settings. This personalized eHealth solution is designed to visualize estimated risk, support shared decision-making, provide guidance on interventions, and monitor adherence to medical and lifestyle interventions.

Our primary aim is to assess the effectiveness of our CVRM eCoach approach in the primary prevention of ASCVD. Additionally, our study will qualitatively evaluate the CVRM eCoach approach with a 1-time semistructured interview with a selection of the participants and the practice nurses. The primary endpoint of the trial is the change in calculated 10-year ASCVD risk and its risk-contributing risk factors 6 months into the CVRM eCoach approach. Secondary endpoints encompass health-related quality of life, user experience, and health care satisfaction through questionnaires.

## Methods

### Study Design

This trial adopts a multicenter, pretest-posttest interventional design. Commencing in April 2024, the study will continue until the completion of recruitment, anticipated to occur in 2025. Recruitment will take place across at least 4 GPs’ offices and 2 hospitals situated in the southern region of the Netherlands. Potential participants are recruited from a pool of individuals registered at the participating GPs’ offices, who are routinely invited to see the practice nurse as part of the primary prevention of ASCVD. Potential participants are also recruited at the cardiac emergency department or outpatient clinic at the hospital, in case no ASCVD is diagnosed. Approval of the study was obtained from the Medical Ethics Committee Zuyderland-Zuyd (02/02/2024, NL84584.096.23, METCZ20230114).

### Study Population

The study population comprises apparently healthy individuals (without a history of diabetes mellitus or ASCVD), aged 40-70 years, who are eligible for CVRM at the GPs’ offices or hospitals. Individuals eligible for CVRM have modifiable risk factors (eg, smoking, hypercholesterolemia, or hypertension) that elevate the risk of ASCVD and are then routinely invited and treated per the Dutch guidelines for CVRM [15]. Eligible individuals must be Dutch-speaking and possess hardware such as a smartphone, computer, or tablet with internet access, along with adequate digital literacy to operate these devices. Exclusion criteria include language barriers preventing sufficient Dutch comprehension and physical, psychiatric, or neurological conditions that hinder completion of study procedures. In addition to the apparently healthy individuals, practice nurses involved in the study are considered part of the study population, as their experiences with using the eCoach will also be included in the qualitative data collection.

### Recruitment

**Figure 1:**
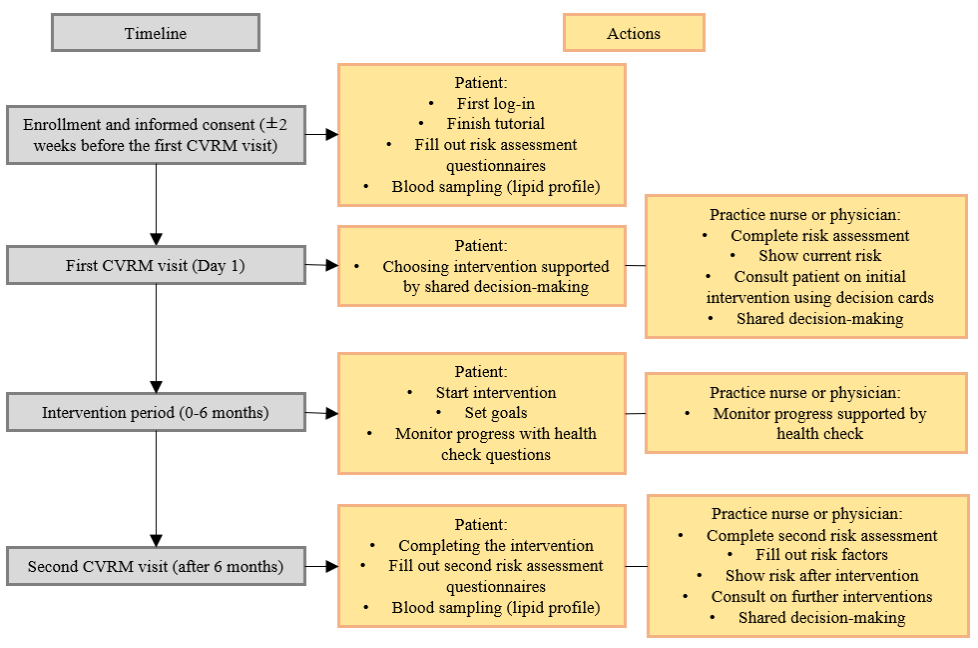
Flowchart of the study and the cardiovascular risk management eCoach approach. CVRM: cardiovascular risk management.

Individuals with an upcoming CVRM consultation at their GP’s office will undergo eligibility screening by the practice nurse. Eligible individuals will receive a preliminary contact by phone, preferably 2 weeks before their scheduled appointment. Individuals at the cardiac emergency department or outpatient clinic will undergo eligibility screening by their treating physician. Individuals screened from the hospital will have their CVRM consultation with one of the physicians from the hospital affiliated with the study (refer to Figure 1 for the study flowchart). Those expressing interest in participation will receive detailed study information from the research team. Upon obtaining written informed consent, eligible individuals will be enrolled in the trial and given login credentials to the eCoach.

### The CVRM eCoach Approach

The eCoach is accessible via smartphone, tablet, or desktop computer. Each participant has their own account and platform interconnected to a separate platform accessible to their practice nurse or physician. In these platforms, participants and practice nurses or physicians can perform the actions as described in the remainder of this section and in Figure 1. A built-in tutorial guides the participant through the platform’s functionalities, and they complete their part of the first risk assessment. This includes information known to the participant, which includes age, gender, zip code for socioeconomic status [16], smoking status, physical activity measured via the Community Healthy Activities Model Program for Seniors (CHAMPS) questionnaire [17], and dietary intake measured via the Eetscore Food Frequency Questionnaire (FFQ) [18].

**Figure 2:**
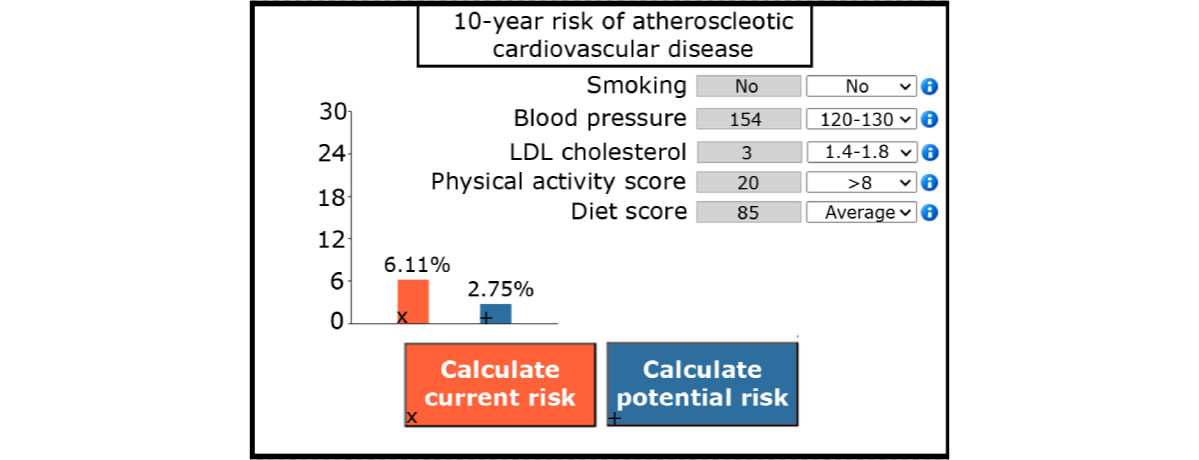
Risk calculation menu (translated from Dutch to English; enlarged and simplified for readability). The risk profile indicates the current (left drop-down menus) and potential risk (right drop-down menus) of atherosclerotic cardiovascular disease, which allows risk comparison after potential targets are achieved. LDL: low-density lipoprotein.

**Figure 3:**
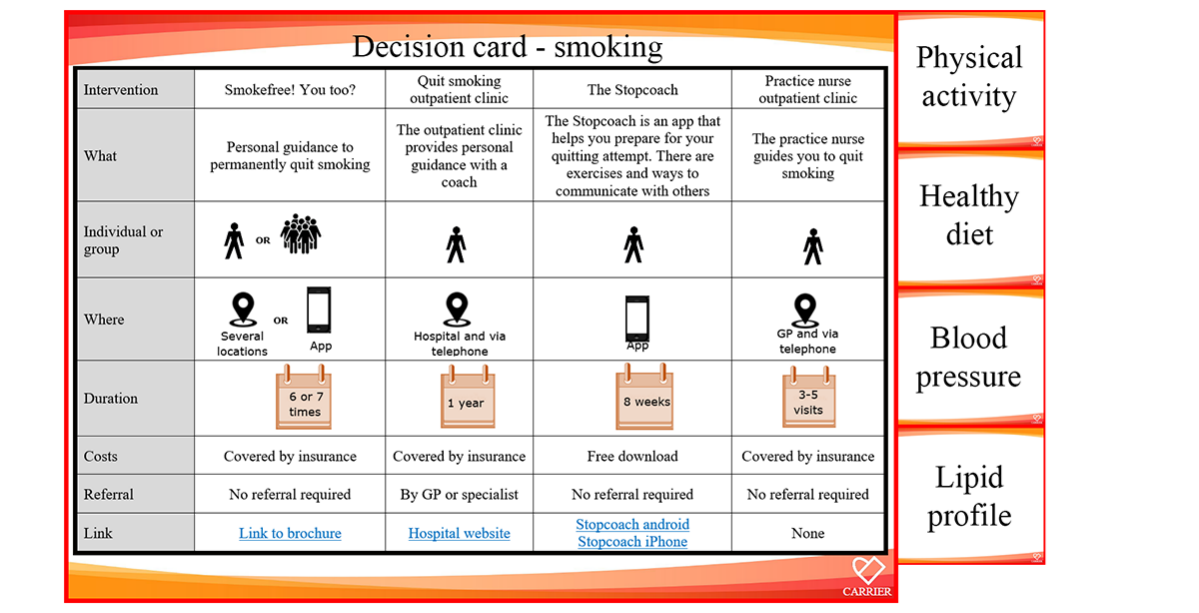
Decision cards (translated from Dutch to English; enlarged for readability). The figure shows an example of a decision card that displays four available smoking cessation interventions. Other decision cards include physical activity, healthy diet, blood pressure, and lipid profile. GP: general practitioner.

At the first CVRM visit, the practice nurse or physician completes the risk assessment by filling out the blood pressure measurement and lipid profile into the eCoach, along with blood pressure medication and lipid-lowering medication. Once completed, the CPM computes the 10-year ASCVD risk, which is graphically depicted in the eCoach dashboard (Figure 2). A drop-down menu is available for each risk factor, allowing participants and practice nurses to interactively view the estimated impact of any interventions, which are displayed as “risk after intervention.” Adjustments can be made for the variables smoking status, blood pressure, low-density lipoprotein cholesterol levels, physical activity, and dietary habits. After risk assessment, the practice nurse and participant can use the “decision cards” offering various lifestyle or medical intervention options to achieve desired goals (eg, smoking cessation assistance from a specialized nurse; Figure 3). Each decision card offers seven attributes of information, which include (1) a short description of the intervention, (2) the group size, (3) the (online) location, (4) the duration, (5) the costs (if any), (6) the necessity for a referral, and (7) a link to provide the first step toward starting the intervention. All interventions are part of routine or existing care, with the eCoach primarily aimed at creating awareness, inciting action, facilitating shared decision-making, monitoring progress, and promoting adherence.

After the initial CVRM visit, participants can use the eCoach to track the evolution of their lifestyle or medical interventions. The eCoach prompts participants with simple weekly inquiries. Examples of inquiries include asking the participant if they picked up their medication (if applicable) or visited the website for their chosen intervention (if applicable). Practice nurses can review responses, which are color-coded as red (requires attention), orange (may require attention), or green (requires no attention). This facilitates early detection of potential challenges the participant faces for their intervention. Practice nurses can encourage participants, when necessary, on the non–content-related aspects of the intervention (eg, remind them to pick up medication or visit the website of the chosen intervention).

**Figure 4:**
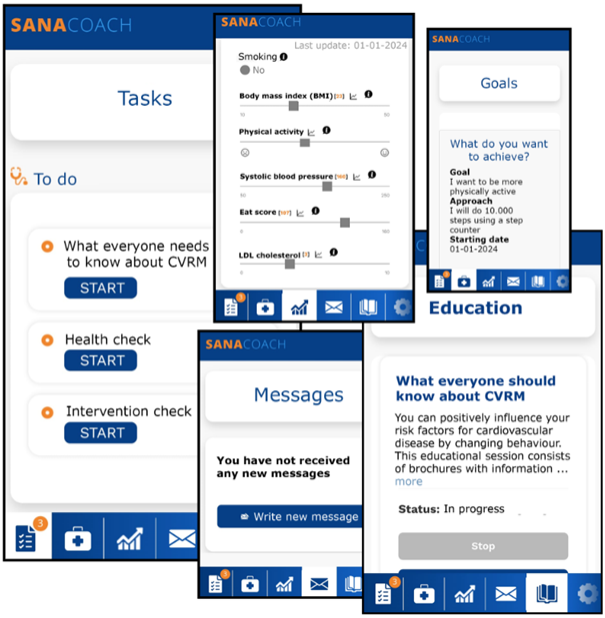
Screenshots of the eCoach (translated from Dutch to English; enlarged for readability). The icons in the bottom row menu, from left to right, represent (1) tasks, (2) goals, (3) dashboard (including risk factors), (4) messages, (5) education, and (6) settings.

Participants can navigate through the eCoach using the menu, depicted with simple symbols at the bottom of the screen (Figure 4). The task menu displays incomplete tasks, including health check questions, educational materials disseminated by the practice nurse, and goals formulated by the participant. The goals menu features short-term and long-term goals, along with previously accomplished objectives. The messaging feature enables participants to communicate with the practice nurse, and vice versa. Practice nurses have the additional capability to broadcast messages to all their participants, such as promoting local health events or sharing informative papers on cardiovascular health. Risk factor statuses are represented on a sliding scale for participants in the eCoach, representing progress compared with previous measurements. The sliders turn green to denote improvement and red to indicate deterioration. Using the concept of shared decision-making between the participant and caregiver, an intervention domain and corresponding strategy can be selected to contribute to ASCVD risk reduction.

Around 6 months into the CVRM eCoach approach, participants undergo a second and last CVRM visit, during which the practice nurse provides consultation on the progress of their 10-year cardiovascular risk profile. This involves a risk reassessment identical to the first visit.

### Clinical Prediction Model

We developed the CPM [19] that estimates baseline 10-year ASCVD risk based on a set of conventional risk factors, individual characteristics, and medication. The complete list of predictors of the baseline risk model is age, gender, socioeconomic status, systolic blood pressure, use of blood pressure medication, total cholesterol, high-density lipoprotein (HDLs), use of lipid-modifying agents, smoking status, moderate to vigorous physical activity (as measured using the CHAMPS questionnaire [17]), and diet (using the Eetscore FFQ [20]). In addition, the model is a prediction under an intervention model [21] that can estimate the effect of combinations of hypothetical lifestyle interventions such as lowering high blood pressure and increasing exercise.

We used the concordance index (C-index) to assess the discriminative performance of the model, which we internally validated using 10-fold cross-validation. The resulting model based on a complete case analysis using the follow-up data available at the time of development achieved a mean C-index of 75.7 (95% CI 65.8-85.6) in the validation sets.

We developed competing risk models using the Fine and Gray [22] method with data from the Maastricht Study [23], a population-based cohort study including approximately 9000 individuals. To create the ASCVD outcome and the competing risk outcome (eg, mortality due to a different cause), we enriched the data from the Maastricht Study with data from Statistics Netherlands and hospitalization data from the Dutch Hospital Data Foundation [24-27]. To estimate the effect of lifestyle interventions, the parametric g-formula, as described by Hernán and Robins [28], was used. Variable selection was driven by expert consensus on relevant confounders, predictors, and the lifestyle interventions targeted by the CARRIER project (eg, smoking cessation, physical exercise, and diet).

In terms of the effect of lifestyle interventions estimated by our model, the hazard ratios (HRs) for smoking cessation and diet improvement were in line with published literature. Our model estimated an HR of 0.47 linked to smoking cessation. A meta-analysis by Mons et al [29] reported an HR of 2.07 (comparing current smokers to never-smokers), which is inversely comparable with the HR in our model. Our model estimated an HR of 0.96 for diet improvement with an increase of 1 SD in the Dutch Healthy Diet Index, equal to the HR of 0.96 estimated by Struijk et al [30]. However, the effect of physical activity estimated by our model was negligible. This might be due to measurement errors related to self-reported physical activity compared with actual physical activity. To address this, we used the HR published by Ramakrishnan et al [31] on a study using accelerometer-measured physical activity on UK Biobank participants. In addition, our model included an HR of 0.80 for each 10-mmHg systolic blood pressure reduction [32] and an HR of 0.79 for each 1 mmol/L reduction in low-density lipoprotein [33]. This is in line with the risk estimation applications supported by the European Society of Cardiology (U-Prevent).

### Measurements

Quantitative data are collected via online questionnaires, blood sampling, and physical examinations at baseline (T0), 3 months (T1), and 6 months (T2) after baseline. The primary outcome includes the change in estimated 10-year ASCVD risk 6 months after using the CVRM eCoach approach as measured by the CPM. The CPM includes age (years), sex (male or female), socioeconomic status, lipid profile (HDL and total cholesterol), blood pressure (in mmHg), lipid-lowering and blood pressure–lowering medication (yes or no), smoking status (current or ex or never), physical activity as measured with the CHAMPS (moderate to vigorous physical activity in hrs/wk) [17], and dietary status (0-160 score) according to the Eetscore FFQ [18,20].

Secondary outcomes encompass the presence of cardiovascular risk factors (smoking, hypertension, hypercholesterolemia, physical activity, and diet) that are used for the CARRIER CPM, health-related quality of life measured with the EQ-5D-5L [34], and usability as measured with the system usability scale [35]. In addition to relevant demographics at baseline, such as living situation (single or living together, with or without children, with or without partner), highest education achieved (eg, primary school, high school, and university), and working status (retired or unemployed or employed + hours worked per week), two 6-item questionnaires are sent out to participants to test digital literacy and satisfaction with health care.

Moreover, qualitative data regarding usability and feasibility are gathered after completing the 6-month follow-up, for which participants and practice nurses are requested to participate in a 1-time interview to evaluate the CVRM eCoach approach. The topics of the interview include (1) the CPM and risk prediction, (2) communication features, (3) user interface, (4) content, (5) time consumption, and (6) health awareness. Interviews will be audio-recorded, and answers to the questions will be annotated fully, clustered, and reported within the previously mentioned topics. We will be using a thematic analysis approach [36].

### Sample Size Calculation

Previous research [37] showed a treatment effect of –2.7 (95% CI –3.3 to –2.2) on the 10-year risk of ASCVD in high-risk participants using mobile technology and smart devices in addition to usual care treatment. The risk score before and after treatment with the mobile technology and smart devices was 14.6 (95% CI 9.5-21.2) and 13.4 (95% CI 8.6-18.2). However, differences exist as our study focuses on primary prevention with likely lower-risk participants (<10% 10-year ASCVD risk) and a more comprehensive risk model including modifiable factors such as diet and physical activity. We assume an SD of 4.5 for the treatment effect and a precision or margin of error of 1. The sample size is calculated using the formula shown in 
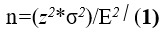
, where *z* is the *z* score (1.96) associated with the chosen 95% CI, σ is the estimated SD (4.5), and E is the margin of error. This results in a sample calculation of 78 participants, adjusted to 100 to account for potential missing data, with replacements considered for early dropouts.

### Statistical Analysis

Descriptive statistics will be used to report baseline characteristics. A paired sample *t* test will be performed for at least the 10-year ASCVD risk, assuming normality. Individual components, including systolic blood pressure, use of blood pressure medication, total cholesterol, HDL, use of lipid-modifying agents, smoking status, and moderate to vigorous physical activity, will be evaluated using the McNemar test for dichotomous outcomes and the paired sample *t* test for continuous variables. An ANOVA analysis will be performed at least on the Eetscore FFQ results and CHAMPS questionnaire results. A *P* value of .05 or less will be considered significant in all analyses.

### Ethical Considerations

The Medical Ethics Committee Zuyderland-Zuyd has approved the study protocol before the start of the study (METC20230114). All participants are required to sign informed consent before participating in the study. Data are de-identified. Participants will be reimbursed for travelling costs.

## Results

**Figure 5:**
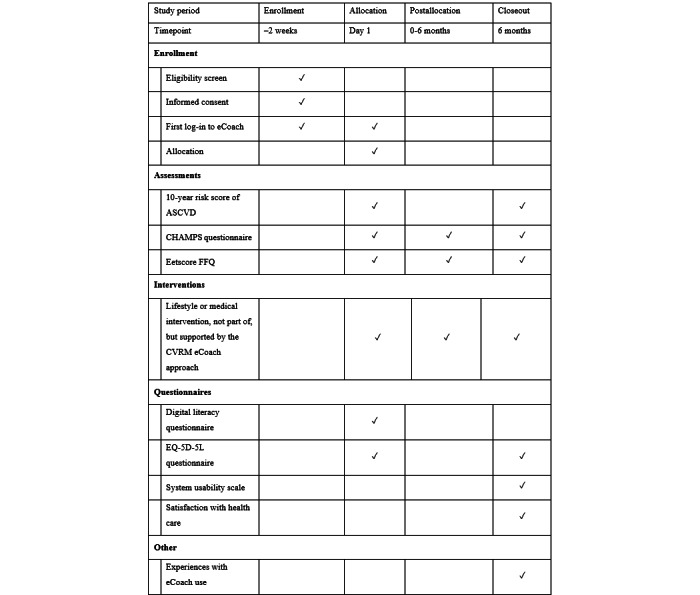
Study overview for the cardiovascular risk management eCoach approach. ASCVD: atherosclerotic cardiovascular disease; CHAMPS: Community Healthy Activities Model Program for Seniors; FFQ: Food Frequency Questionnaire; CVRM: cardiovascular risk management.

Recruitment began in March 2024 and will continue until 100 participants have been recruited, which is expected in 2025. A full overview of the study can be found in Figure 5.

## Discussion

### Principal Findings

This manuscript outlines the study protocol for a multicenter, pretest-posttest interventional trial investigating the impact of our CVRM eCoach approach on reducing the estimated 10-year ASCVD risk in apparently healthy individuals. The eCoach was developed through a cocreation process involving participants, caregivers, health care providers, eHealth developers, and researchers. This collaborative approach aimed to tailor the intervention to align with the individual needs and preferences of participants while also considering the daily practices of involving primary prevention. The final personalized eCoach includes several key features: (1) a risk assessment based on a newly developed CPM that incorporates personalized risk factors and lifestyle variables, (2) a dashboard that visualizes personalized risk and facilitates risk communication by showing both current risk and potential reductions through hypothetical interventions, (3) intervention decision cards to help participants and health care providers in selecting interventions tailored to the participant’s specific characteristics, and (4) an adherence monitoring to track participant’s compliance with the selected interventions.

The CPM has been developed by the CARRIER consortium on data from the Maastricht Study, enriched by data from Statistics Netherlands and hospitalization data from the Dutch Hospital Data Foundation [24-27]. By using artificial intelligence, the model is constructed to estimate ASCVD risk in apparently healthy individuals [14] and trained on data from a cohort of Dutch patients enrolled in the Maastricht Study, a local cohort study [23]. In selecting variables for the CPM, we prioritized those that are readily available or easily obtainable during routine CVRM visits. Therefore, this model incorporates not only conventional risk factors but also publicly available socioeconomic data through the participant’s zip code [16], along with data on physical activity and dietary habits, using validated questionnaires (even though these might be prone to recall and social desirability biases). By integrating lifestyle factors into the models and dashboard, we aim to improve the precision of risk estimation while also highlighting potential risk reduction for participants and encouraging greater adherence to recommended lifestyle goals. To ensure feasibility in primary care settings, we chose not to include data from advanced imaging techniques such as coronary imaging [13,38,39] or additional laboratory measurements such as proteomes [40] and lipoprotein a [13], despite their known potential to improve risk estimation. Future steps for the CPM include model validation, model comparison to established risk models (eg, SCORE2), and a model impact analysis. In an ideal situation, data from existing users allows for the model to be updated as well.

The dashboard with personalized risks needs to create awareness and incite action. Effective risk communication is pivotal in prevention, as it bridges the gap between medical knowledge and public understanding. This involves conveying the actual meaning of personalized risks (being more personalized but still only risks that may not be realized), the urgency to change lifestyle behavior (even if figures seem to be rather small), and showing potential benefits (that are relevant to the individual participant). A combination of different communication formats, for example, percentages and visuals, is advisable [41]. In this case, the dashboard can be used as a starting point for the conversation. Essential is conveying the information meeting the needs of the target population [42] to improve health literacy. Tailoring communication strategies to account for an individual’s cultural, social, and educational background might further enhance receptivity and adherence to preventive measures.

Numerous medical and lifestyle interventions are available to reduce the risk of ASCVD; yet, many individuals are unaware of their options or lack sufficient knowledge about the pros and cons of each alternative. This often results in uninformed decisions that do not align with the individual’s circumstances and needs [43]. A personalized care approach can help health care professionals guide apparently healthy individuals in choosing and adhering to suitable lifestyle interventions [44]. Decision support tools, such as patient decision aids and decision cards [45], are emphasized when discussing available treatment alternatives. These tools provide balanced and neutral information on relevant options, helping patients make informed health care decisions. Decision aids provide in-depth information and support complex decision-making processes with detailed explanations and interactive features. In contrast, decision cards are typically concise and visually straightforward, often presented on a single card, and function as a quick-reference tool for specific choices or actions. In the CVRM eCoach approach, 5 decision aid cards were integrated for the key intervention domains, including smoking, physical activity, diet, blood pressure, and lipid profile. These cards were created by the study team, tested with end users, and customized to fit our regional health care context.

Achieving durable behavior change is essential for reducing the risk of future cardiovascular diseases. Our study incorporated several behavior change techniques to promote long-term adherence, including self-monitoring, performance feedback, goal setting, information on the consequences of behaviors, and guidance on how to perform the desired behaviors [46]. Research suggests that combining eHealth interventions with face-to-face contact can significantly enhance adherence [47]. To further support adherence, our practice nurses and physicians are available throughout the 6-month follow-up period to provide monitoring, support, and answers to any questions participants may have.

### What Do We Expect to Learn From This Study?

The primary outcome of this study is the change in estimated 10-year ASCVD risk from baseline to 6 months after the initiation of the eCoach solution. While the ultimate goal is to reduce actual ASCVD events, such outcomes require long-term follow-up, which was not feasible at this stage. Therefore, our focus is on reducing the estimated risk, with the expectation that this reduction will ultimately translate into a decrease in actual events, an increase in community health, and a decrease in health care costs on a population level [48]. Moreover, we aim to address individual risk factors, enhance health-related quality of life, assess user experience, and evaluate health care satisfaction. Insights gained from these latter 2 endpoints may provide valuable feedback for refining future CVRM decision-making processes for interventions tailored to this population.

### Future Perspectives

In light of constrained financial and human resources, innovative solutions are increasingly imperative to augment our health care services [49,50]. Future CPMs may be fully integrated with hospital electronic health records and automatically extract relevant data from them, as well as wearable devices, integrating longitudinal laboratory results, vital signs, physical activity levels, socioeconomic factors, and environmental variables. This comprehensive data collection has the potential to enhance the early detection of individuals at risk for ASCVD.

High-risk individuals will receive personalized lifestyle advice to mitigate their risk. When medication is necessary for risk reduction, patients will be referred to health care professionals for further discussion and guidance. This process will be supported by eHealth technologies and fully integrated patient decision aids, tailored to individual patient characteristics for CVRM. To monitor chosen interventions, wearable devices will be used to gather unique patient information about lifestyle, ensuring assessment and adjustment of personalized health strategies.

The CARRIER consortium is actively developing data infrastructures and ethical and legal frameworks for responsible data handling in health care. We envision a future in health care that is data-driven and bolstered by digital health solutions.

### Conclusion

The CARRIER consortium is actively developing data infrastructures, as well as ethical and legal frameworks, for responsible data handling in health care. We envision a data-driven future in health care, supported by digital health solutions.

## Appendix

Multimedia Appendix 1 SPIRIT (Standard Protocol Items: Recommendations for Interventional Trials) checklist.

## Abbreviations

ASCVD: atherosclerotic cardiovascular disease
CARRIER: Coronary Artery disease: Risk estimations and Interventions for prevention and Early detection
CHAMPS: Community Healthy Activities Model Program for Seniors
CPM: clinical prediction model
CVRM: cardiovascular risk management
FFQ: Food Frequency Questionnaire
GP: general practitioner
HDL: high-density lipoprotein
HR: hazard ratio
SCORE2: Systematic Coronary Risk Evaluation 2

## Data Availability

The datasets are generated during the study. Once completed, they can be obtained from the corresponding author upon reasonable request.
